# Innate immune recognition and immunomodulatory effects of bacteriophages: implications for phage therapy

**DOI:** 10.3389/fimmu.2026.1851108

**Published:** 2026-06-11

**Authors:** Tomoko Hanawa, Mayuko Tanaka, Debora Satie Nagano, Ken Shirato, Sanae Hasegawa-Ishii, Takeaki Matsuda

**Affiliations:** 1 Department of General Medicine, Kyorin University School of Medicine, Tokyo, Japan; 2 Department of Hygiene and Public Health, Kyorin University School of Medicine, Tokyo, Japan; 3 Faculty of Health Sciences, Kyorin University, Tokyo, Japan; 4 Department of Traumatology and Critical Care Medicine, Kyorin University School of Medicine, Tokyo, Japan

**Keywords:** bacteriophage, immunomodulatory, innate immunity, phage therapy, *Staphylococcus aureus*

## Abstract

Bacteriophages (phages) have been explored as a potential treatment for infectious diseases owing to their bacteriolytic ability. However, accumulating evidence indicates that phages interact with the host immune system and modulate innate immune responses. Phage particles, genomes, and phage-derived components are sensed by pattern recognition receptors, including Toll-like receptors and cytosolic nucleic acid sensors, leading to context-dependent activation or suppression of inflammatory signaling pathways, such as nuclear factor κ-light-chain-enhancer of activated B cells and type I interferon responses. In clinical settings and *in vivo* experiments, phage-induced immunomodulation influences infection outcomes by promoting bacterial clearance, limiting excessive inflammation, and facilitating tissue repair. However, phage-mediated immune regulation is not uniformly beneficial and may impair antibacterial immunity or exacerbate inflammatory diseases in certain contexts. In this review, we summarize current knowledge of phage innate immune recognition, the immunomodulatory effects in infectious and inflammatory settings, and the implications of these interactions for the safety and efficacy of phage therapy. A deeper understanding of phage–immune system interactions is essential for optimizing phage selection and administration. This knowledge can facilitate the development of combination therapies tailored to the host immune status and specific disease contexts.

## 1 Introduction

The concept of phage therapy, which utilizes the lytic activity of bacteriophages (phages) to treat bacterial infectious diseases, was proposed soon after the discovery of phages but has been the subject of debate for over a century (1). Subsequently, research in this field declined with the advent of antibiotics (1). However, with the increasing incidence of multidrug-resistant (MDR) bacterial infections, the efficacy of phage therapy, which eradicates pathogens through mechanisms distinct from those of antibiotics, has been re-evaluated and is now considered a promising treatment option (2–7).

The primary focus of phage therapy is the lytic activity of phages against bacteria. Re-evaluation of their therapeutic potential, particularly in the context of antibiotic resistance, has increasingly highlighted the significant influence of phage–host immune interactions on clinical outcomes (8–13). Crucially, phages interact with eukaryotic cells, such as human cells, through phagocytosis or endocytosis (14), eliciting both innate and adaptive immune responses (3, 15–18). However, the precise mechanisms through which phages modulate the immune system remain unclear. Therefore, understanding the host immune response to phages and the complex interplay among host immunity, pathogenic bacteria, and the therapeutic effects of phages is essential to fully understand the effect of phage therapy on clinical outcomes (19).

Recent comparative studies have suggested that herpesviruses may have branched off from a subclass of *Caudovirales* (possibly from the *Myoviridae* family or one of its subfamilies) (20). *Caudovirales*, unlike *Herpesviricetes*, lack envelopes and teguments; however, their capsids share structural similarities (21). Moreover, tailless double-stranded DNA (dsDNA) phages and certain eukaryotic dsDNA viruses share a major capsid protein with a double-jelly roll structure (22). These evolutionary roots and structural similarities may serve as a reference for comparison to understand phage-mediated immunomodulatory mechanisms (23).

Pathogenic bacteria exhibit diverse infection strategies and host-specific immune responses (24). In phage therapy, phages are introduced after the host immune system has begun responding to bacterial infection. Consequently, it is important to evaluate not only the host immune response to phages but also the effect of phages within the context of an immune response already triggered by bacterial infection.

By integrating insights from comparative viral immunology and the effects of phages observed *in vivo* experimental models and clinical studies, we investigate the immunomodulatory role of phages in clinical settings. This approach facilitates discussion of the effects of phages on therapeutic efficacy and safety, and outlines future challenges and directions (2, 25).

## 2 Recognition of eukaryotic viruses and phages by innate immunity and downstream reactions

Interactions between the host and pathogens begin with innate immunity via pattern recognition receptors (PRRs), which are sensors that allow the host to recognize pathogen-associated molecular patterns (PAMPs). As phages also contain structures equivalent to PAMPs, they are recognized by host PRRs, which can initiate the activation of the type I interferon (IFN) and nuclear factor κ-light-chain-enhancer of activated B cells (NF-κB) signaling pathways (22), further eliciting downstream antiviral reactions and inflammation.

### 2.1 Recognition of phage genomes in endosomes

**Figure 1:**
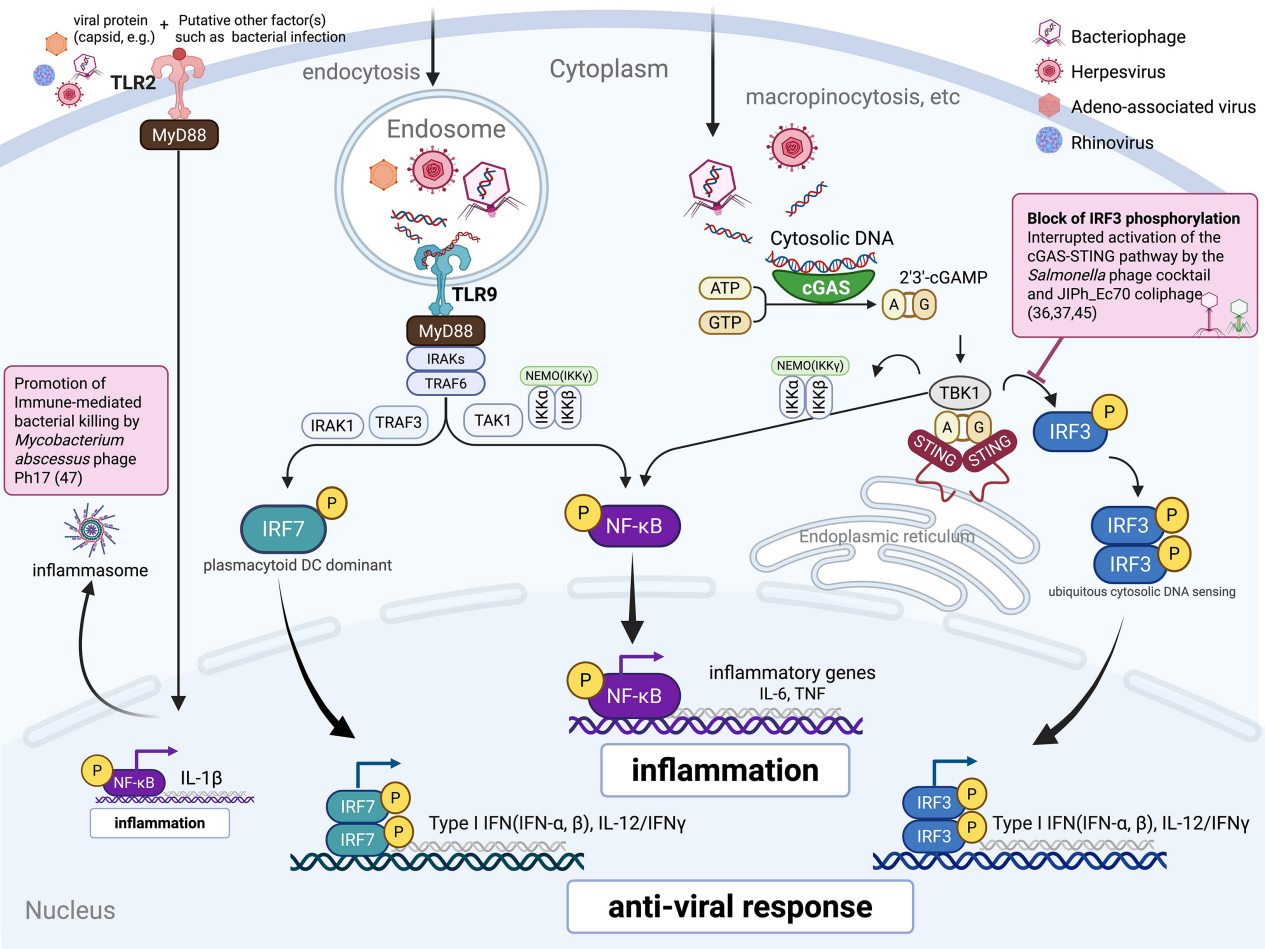
Innate immune response via recognition of viral genomic DNA or proteins and immune modulation by bacteriophages affecting the TLR and cGAS–STING pathways. Viral genomic DNA are recognized by TLR9 in the endosomes and by cGAS, which synthesizes 2′-3′-cGAMP to activate STING in the cytosol. Following activation of the MyD88–NF-κB system and the interferon regulatory factor (IRF) system, inflammation and antiviral responses are induced. Similar to viral DNA in eukaryotes, phage genomic DNA are recognized by TLR9 and cGAS. Additionally, TLR2 on the surface of cells recognizes capsid proteins or other viral proteins and induces inflammation and antiviral responses (22). Certain bacteriophages modulate these pathways; the *Salmonella* phage cocktail (31, 32) and JIPh_Ec70 coliphage (33) suppress IRF3 phosphorylation and interfere with cGAS–STING signaling. *Mycobacterium abscessus* phage Ph17 promotes the bactericidal activity of macrophages, suggesting that recognition via TLR2 on the surface of macrophages and activation of NLRP3 forms an inflammasome (34). Created in BioRender.

Viral infections are detected by recognizing viral genomic nucleic acids within endosomes. Specifically, double- and single-stranded RNAs and non-methylated CpG DNA motifs are detected as PAMPs by the Toll-like receptors TLR3, TLR7/8, and TLR9, respectively (26–29). These interactions within the endosomes of macrophages and dendritic cells (DCs) trigger inflammatory responses via NF-κB activation and antiviral responses via type I IFN signaling (30) (**Figure 1**). Considering these host innate immunity mechanisms, it is essential to explore whether PRRs recognize phage genomes, particularly in light of their established roles in sensing viral nucleic acids (35–37). Based on this conjecture, the recognition of phage DNA by TLR9 within the endosomes has been demonstrated, resulting in the activation of downstream reactions. This also resulted in the production of interleukin (IL)-12 and IFN-γ using phages of *Bacteroides thetaiotaomicron* and *Lactobacillus plantarum*, as well as a cocktail consisting of NC-A, NC-B, and NC-G which are specific to adherent-invasive *Escherichia coli* (38). Furthermore, a *Salmonella*-targeting phage cocktail consisting of vB_SenM-2, myovirus (39) and vB_Sen-TO17, siphovirus (31) is recognized by TLR9 and activated MyD88, followed by activation of IRF7 and NF-κB in chicken blood cells (32). Further supporting this mechanism, the transfection of these phage DNAs into chicken lymphoblasts (DT40 cells) can activate the TLR9 pathway (32) (**Figure 1**).

### 2.2 Recognition of phage genomes in the cytoplasm

In addition to endosomal recognition, cytosolic PRRs are crucial for detecting viral nucleic acids. For instance, RNA structures, such as RNA virus genomes or transcripts of DNA viruses, which are absent in mammalian cells, are recognized by RIG-I-like receptors in the cytoplasm (40–42). This activates both NF-κB and interferon regulatory factors (IRF)3 or IRF7. Consequently, type I IFN and NF-κB trigger inflammation and antiviral response. For DNA viral genomes, circular GMP-AMP synthase (cGAS) induces the transcription of type I IFN (IFN-β) via the transcription factor IRF3 (43, 44). cGAS recognizes DNA generated by reverse-transcribed retrovirus genome and damaged host dsDNA, promoting inflammatory responses through the cGAS–STING–NF-κB pathway (43, 45, 46).

The phage cocktail targeting *Salmonella*, consisting of vB_SenM-2 and vB_Sen-TO17, is also sensed by cGAS in addition to TLR in endosomes, leading to the activation of the cGAS–STING pathway (32). Furthermore, the DNA of JIPh_Ec70, a myovirus of *E. coli*, also activates STING, although recognition by cGAS has not been verified. However, a similar response has not been observed with the *Klebsiella pneumoniae* phage JIPh_Kp127, probably due to differences in cellular uptake efficiencies (33). Therefore, differences in phage structure and intracellular localization are likely to influence the resulting immune responses.

### 2.3 Recognition of viral proteins by TLR2

Multiple proteins from eukaryotic viruses, such as rotavirus, herpes simplex virus, rhinovirus, and adenovirus activate TLR2 (22). The capsid of the adeno-associated virus vector (AAV), a non-enveloped single-stranded DNA (ssDNA) virus, induces NF-κB-dependent production of inflammatory cytokines via TLR2; however, a type I IFN response is not induced (47). Unlike pathogenic viruses, AAV does not replicate independently or damage cells. In this regard, AAV shares similarities with phages.

In recent years, phage capsids that can be recognized by PRRs have been reported (22). Activation of the NLRP3 inflammasome and induction of IL-1β production are triggered via TLR2 signaling by recognition of the Ph17 phage protein, resulting in the suppression of intracellular survival of *Mycobacterium abscessus* (34). However, when Ph17 was administered to macrophages alone, a similar response was not observed (34), indicating that the stimulation of TLR2 by phages likely requires additional triggers, such as bacterial infection.

## 3 Immune modulation by eukaryotic viruses and phages

### 3.1 Regulation of NF-κB signaling

The transcription factor NF-κB is a pivotal mediator of inflammatory responses. The regulation of NF-κB signaling is critical for both viral and phage-mediated immune evasion. Many pathogenic viruses dynamically regulate NF-κB signaling depending on the stage of infection to colonize or maintain infection. While NF-κB suppression contributes to immune evasion and persistent infection by reducing inflammation and inhibiting apoptosis (48, 49), its activation can also be advantageous for viral spread by modulating antiviral responses. Pathogenic viruses activate the NF-κB pathway through distinct, often highly specialized mechanisms that have evolved across viral lineages. Although viral subfamilies (or genera) tend to have characteristic mechanisms for inhibiting NF-κB, homologous inhibitory proteins or mechanisms shared across them have also been reported (50).

In light of the similarity of strategies based on the genetic background of eukaryotic viruses, phages belonging to the same genus might show commonality of immune modulation effects. Transcriptome analysis has revealed an anti-inflammatory profile for all phages in A549 epithelial cells (51). In contrast, siphoviruses (CK02) and podoviruses (CK21) elicited a mild inflammatory response, whereas the myovirus phage (CK12) maintained a pro-inflammatory response in macrophages (51). JIPh_Ec70 activated the NF-κB pathway, resulting in strong induction of IL-1β and IL-6 expression, whereas JIPh_Kp127 did not activate the pathway, suggesting low phagocytosis efficiency (33). The T4 phage, a representative myovirus considered highly immunogenic (52), induces little to no NF-κB activity or production of inflammatory mediators in various cells (33, 53, 54).

### 3.2 Regulation of the cGAS–STING pathway

Viruses have diverse strategies to inhibit cGAS–STING signaling and evade the immune system (43). The tegument proteins of herpesviruses directly bind to the catalytic domain of cGAS, inhibiting its dsDNA-binding capability and enzymatic activity, thereby suppressing the STING pathway (55). Additionally, the capsid protein UL38 is recognized by cGAS; however, it inhibits the TBK1-IRF3 signaling pathway by serving as a STING antagonist (56). These findings demonstrate the diversity of DNA sensor evasion strategies employed by herpesviruses (56).

Although the recognition of cytosolic DNA by cGAS–STING mediates IRF3 activation, some phages exhibit distinct responses. Intriguingly, JIPh_Ec70 seems to activate this pathway via STING; however, IRF3 is not phosphorylated, resulting in the activation of the NF-κB pathway in peripheral blood mononuclear cells (33). A *Salmonella* phage cocktail consisting of vB_SenM-2 and vB_Sen-TO17 blocks the phosphorylation of IRF3 under cGAS–STING pathway induction, resulting in the significant suppression of inflammation (32). Podlacha et al. hypothesized that this response, characterized by the suppression of IRF3 phosphorylation, occurs because phage DNA is not an efficient substrate for RNA polymerase III, resulting in insufficient double-stranded RNA production (32). Furthermore, this may explain why the suppression of IRF3 phosphorylation by the cocktail in chickens infected with *Salmonella* reduces the number of viable bacteria to the same extent as antibiotics and suppresses inflammation more strongly, leading to a marked improvement in symptoms (57). Consequently, the downstream responses likely vary by phage species and may reflect phage-specific immune responses. However, the suppression of IRF3 phosphorylation may be a response to phages.

## 4 Effect of phages under activation of immunity by various stimuli

Recent studies have demonstrated the direct immunomodulatory potential of specific phages. However, explaining the clinical effects of phages remains challenging owing to the complexity of host–pathogen immune interactions. Focusing on well-characterized infection models is often more beneficial. Given the clinical urgency of combating MDR pathogens, we focus on *Staphylococcus aureus* infection as a well-characterized model.

### 4.1 Pathogenicity of *S. aureus* and its phage therapy

Despite being a commensal bacterium colonizing one-third of the human population, *S. aureus*, a Gram-positive facultative aerobic coccus, can cause various infectious diseases such as skin and soft tissue infections, bone and joint infections, chronic rhinosinusitis, and pneumonia (58, 59). In some cases, it can cause life-threatening infections, such as bloodstream infections and sepsis (58, 60, 61).

The pathogenicity of *S. aureus* is due to its ability to modulate host immunity, depending on the stage of infection. During the acute phase of infection, numerous toxins that induce excessive inflammation surpass the immune response to PAMPs and decrease the killing ability of phagocytic cells. As the infection proceeds, *S. aureus* forms biofilms that exhibit low metabolism and secrete extracellular matrix components, such as DNA, RNA, polysaccharides, and toxins, by altering gene expression, leading to a physiology different from that of planktonic cells. Extracellular matrixes protect bacterial cells as a physical barrier from attack by immune cells and reduce inflammation response. Inflammation is suppressed by neutrophil killing by toxins (62) or by modulating the immune system by biofilm-associated molecular patterns or other factors contained in the biofilm matrix (63–65) to establish infectious lesions and progress to chronic infection. Adaptive immunity, including antibody production, is suppressed by protein A (SpA) of *S. aureus* (65, 66). Binding of SpA to the Fc region of IgG inhibits opsonization and phagocytosis, promotes B cell receptor crosslinking, and induces B cell apoptosis (60). In addition, SpA-IgG complexes can cause leukocyte necrosis and leukotoxicity (67). Additionally, biofilms contain persisters, which temporally stop or diminish growth to survive under various deleterious conditions, such as the presence of antibiotics (68). Therefore, biofilms are resistant to antibiotics (69–71).

Wound and surgical site infections and device-related infections are often caused by *S. aureus*. Antibiotic therapy is often limited to *S. aureus* infections because of its highly strategic virulence factors. Phage therapy offers many advantages in this context. By utilizing polysaccharides, such as wall teichoic acid, as receptors to infect bacterial cells, *S. aureus* phages can infect multiple strains in a broad host range (72–75). Additionally, certain phages can disrupt biofilms by degrading their matrix using depolymerases (76, 77); therefore, immunomodulation is negated, and antibiotics become effective again. The biofilm-disrupting capabilities of phages improve the effectiveness of therapies for joint replacement surgeries and biofilm-associated diseases (78, 79). Phages can be directly applied to infection sites to enhance therapeutic efficacy. These phage therapy factors are also beneficial for improving chronic rhinosinusitis by disrupting biofilms and exerting immune-modulatory effects (80, 81). Accordingly, clinical application of phage therapy for *S. aureus* infections is increasing (82–84).

### 4.2 Immune response to phage therapy in an *in vivo* model of *S. aureus* infection

In some cases, phages against *S. aureus* infections are effective in reducing bacterial load and suppressing inflammation (8–13). However, the host immune response to phage administration, such as changes in immune cell composition and cytokine levels, has not yet been analyzed. Therefore, it remains unclear whether the improvement is due to a reduction in the bacterial count caused by phages or the modification of the immune response.

Instead, the suppression of inflammation by phage administration has been shown through *in vivo* experiments. Ghanaim et al. analyzed the effect of phage therapy on cytokine levels in a rat model of diabetic foot ulcer infection (13). An increase in anti-inflammatory marker levels (IL-10 and IL-4) and a decrease in inflammatory cytokine levels [tumor necrosis factor (TNF)-α, IL-1β, IL-8, and monocyte chemoattractant protein-1] were observed. Notably, phage treatment facilitated collagen deposition and demonstrated histologically superior outcomes compared to antibiotic therapy. A cocktail of AB-SA01 containing three myoviruses (J-Sa36, Sa83, and Sa87) showed equivalent or superior efficacy compared to vancomycin in a mouse model of streptozotocin-induced diabetes (85). However, without data on inflammatory cytokines and the results of histological analyses, it remains unclear whether these phages can suppress excessive inflammation.

Immunomodulatory effects of phages, which are not limited to inflammation, have also been suggested in *S. aureus* infections. In mice that received intranasal administration of phage 536_P1, a slight increase in IFN-γ and IL-12 levels was noted in the lungs; however, similar responses were not observed when the phage was administered to infected mice (86). Furthermore, another myovirus phage phiMR003 with a broad host range among methicillin-resistant *S. aureus* (MRSA) clinical isolates (87) has demonstrated efficacy against MRSA infections. Intriguingly, phiMR003 infects KYMR58, a strain not susceptible to phiMR003 *in vitro*, reducing the bacterial load, accelerating wound healing, and decreasing inflammatory cytokine levels (88). As phiMR003 does not trigger inflammation in mouse peritoneal macrophages and suppresses the expression of lipopolysaccharide (LPS)-induced inflammatory cytokines, immunomodulatory effects, including the reduction of excessive inflammation, improvement of infection, and promotion of wound healing, have been reported (88).

### 4.3 Immune response of cells to *S. aureus* phage

At the cellular level, cytokine production against phages and alteration of the responses in the presence of stimuli, such as LPS, have been investigated. For instance, the myovirus vB_SauM_JS25 reduces mRNA levels of inflammatory cytokines induced by *S. aureus* or LPS by inhibiting NF-κB phosphorylation in MAC-T cells (89). Interestingly, vB_SauM_JS25 induces an antiviral response upon activation by mouse norovirus (90). Furthermore, when human peripheral blood monocytes are stimulated with *S. aureus* phage (ISP, myovirus) together with *Pseudomonas aeruginosa* phages (PNM, LUZ19, podovirus; GE-vB_Pae-Kakheti25, siphovirus; 14-1, myovirus), both inflammatory and anti-inflammatory cytokines are induced. However, an anti-inflammatory response predominates when the response is weak (91). Notably, the addition of LPS increases IL1RN and markedly decreases CXCL1/5 stimulation, further enhancing the anti-inflammatory regulation by ISP and *P. aeruginosa* phages (91).

### 4.4 Immune response to endolysin

Beyond whole-phage applications, the therapeutic potential of phage-derived components has been explored. For example, endolysins are peptidoglycan hydrolases that hydrolyze the cell wall when phages are released after propagation. Gram-positive bacteria, such as *S. aureus*, do not have an outer membrane; therefore, endolysins can lyse bacteria exogenously, making them a potential option for the treatment of bacterial infections (92–95). Given the complexities of whole-phage interactions, exploring phage-derived components offers a more targeted approach for immunomodulation.

Recombinant cysteine- and histidine-dependent amidohydrolase/peptidase domain of the LysK enzyme significantly accelerates healing by suppressing the inflammatory cytokine response and promoting the proliferative phase of wound healing through the bactericidal and other effects of endolysins (95). Furthermore, immunomodulatory effects, such as a reduction in foreign body giant cell and osteogenic cell counts, occur via the immune system (94).

## 5 Anti-inflammatory effects of phages under stimulation with other inflammatory factors

Phage-mediated suppression of inflammation has been observed in response to stimuli other than bacterial infection. A phage cocktail comprising multiple phages suppress the increase in inflammatory cytokines and improve colitis by restoring tight junctions in a model of dextran sodium sulfate (DSS)-induced colitis (96). Similarly, in a mouse allogeneic skin transplantation model, anti-inflammatory effects have been demonstrated through the suppression of NF-κB activation (53).

At the cellular level, T4 phage almost completely suppresses NF-κB activation induced by HSV-1 (53). Conversely, the immunomodulatory effects of phages are not exclusively anti-inflammatory. In mice pretreated with phage cocktails derived from patients with inflammatory bowel disease or phages sourced from gut bacteria, DSS-induced colitis is aggravated in a TLR9-dependent manner (38).

The aforementioned studies collectively highlight the suppressive effects of phages and their derivatives on inflammation and tissue damage. However, to fully evaluate the safety and efficacy of phage therapy, it is essential to investigate beyond the phage’s direct effect and comprehend the immune-mediated effects arising from the intricate interactions among phages, bacteria, and host immune responses.

## 6 Challenges to and prospects of phage therapy

Immunomodulation by phages is increasingly recognized for its significant effects in the treatment of infectious diseases. Notably, phages enhance pathogen elimination through immune cell activation; minimize tissue damage by curbing excessive inflammation, which is likely independent of bacterial load reduction; and expedite wound healing (**Table 1**). These effects are typically not observed with standard antibiotic treatment.

**Table 1:** Immune recognition and cellular outcomes of phages and cocktails described in this review.

Phage or cocktail		Classification^1)^	Host	Effects on immune response	Reference
*Escherichia coli* phage cocktail	NC-A	Podovirus	*E. coli* (adherent-invasive *E. coli*)	TLR9 recognition, followed by IL-12 and IFN-γ production	(38)
	NC-B	Podovirus (lysogenic)			
	NC-G	Myovirus			
*Bacteroides thetaiotaomicron* phage		N/A^2^**^)^**	*B. thetaiotaomicron*	TLR9 recognition, followed by IL-12 and IFN-γ production	(38)
*Lactobacillus plantarum* phage		N/A^2^**^)^**	*L. plantarum*	TLR9 recognition, followed by IL-12 and IFN-γ production	(38)
*Salmonella* phage cocktail	vB_SenM-2	Myovirus	*Salmonella*	Activation of NF-κB via the TLR9–MyD88 pathway and inhibition of IRF3 phosphorylation despite cGAS–STING activation, resulting in an anti-inflammatory response due to interrupted immune signaling (IFNβ is produced, but IFNα is not).	(31, 32)
	vB_Sen-TO17	Siphovirus			
JIPh_Ec70		Myovirus	*E. coli*	cGAS–STING activation; NF-κB activation; strong induction of IL-1β and IL-6; blocked IRF3 phosphorylation	(33)
JIPh_Kp127		Siphovirus	*Klebsiella pneumoniae*	No activation of cGAS–STING	(33)
T4 phage		Myovirus	*E. coli*	No activation of NF-κB and induction of pro-inflammatory cytokines; however, under HSV-1–induced NF-κB activation, NF-κB is suppressed.	(52–54)
Ph17		N/A^2^**^)^**	*Mycobacterium abscessus*	IL-1β production induced by TLR2 recognition, followed by NLRP3 activation, promotes immune-mediated bacterial killing *in vivo*. However, this response is weak when Ph17 is applied alone.	(34)
AB-SA01	J-Sa36	Myovirus	*Staphylococcus aureus*	Antibacterial effect against *S. aureus* (immune response was not assessed)	(13)
	Sa83	Myovirus			
	Sa87	Myovirus			
536_P1		Myovirus	*S. aureus*	Mild induction of IFN-γ, IL-12, and chemokines was observed in the lungs without infection; however, these changes were absent in infected animals.	(85)
phiMR003		Myovirus	*S. aureus*	Decreased inflammatory cytokine levels in mouse infection by phage-insensitive *S. aureus*; suppression of LPS-induced inflammation in macrophages	(87, 88)
vB_SauM_JS25		Myovirus	*S. aureus*	NF-κB phosphorylation inhibition; reduction of inflammatory cytokine mRNA; induction of antiviral response	(88, 89)
ISP		Myovirus	*S. aureus*	Anti-inflammatory cytokine induction; elevated IL-1RN levels in response to LPS	(90)
GE-vB_Pae-Kakheti25		Siphovirus	*Pseudomonas aeruginosa*	Anti-inflammatory dominant response (weak), followed by downregulation of TNF-α	(90)
LUZ19		Podovirus	*P. aeruginosa*	Anti-inflammatory dominant response (weak), followed by downregulation of TNF-α	(90)
PNM		Podovirus	*P. aeruginosa*	Anti-inflammatory dominant response (weak), followed by downregulation of TNF-α	(90)
14-1		Myovirus	*P. aeruginosa*	Anti-inflammatory dominant response (weak), followed by downregulation of TNF-α	(90)
Pf phage		Filamentous phage (*lysogenic, single-stranded DNA phage)	*P. aeruginosa*	TLR3-TRIF pathway activation induces type I IFN secretion, TNF-α production, and suppresses neutrophil mobilization, thereby promoting chronic infection	(97, 98)

^1)^Classification is based on virion morphology.

^2)^Not applicable

Various experimental systems with different cell types or multiplicity of infection and bacteria-derived contaminants can lead to diverse cellular outcomes, underscoring the need for standardized protocols. Ensuring that the observed immunomodulatory effects are phage-derived and not due to contaminants such as endotoxins, as noted in older literatures, is crucial to prevent a misinterpretation of the actual response of phages.

Despite the potential for detection by PRRs, strong immune responses have not yet been observed in response to phages. This low reactivity of phages may stem from their non-replicative nature in human cells and the specific PRR pathways with which they engage, as detailed above. Pathogenic viruses induce a strong innate immune response by releasing damage-associated molecular patterns (DAMPs) associated with cell lysis. Contrastingly, in AAV-based gene therapies, aside from the effects of dosage and pre-existing immunity, activation of the innate immune system is relatively mild (97). Although immunological comparisons should be carefully considered, AAVs, which are similar to phages in their non-replicative nature and lack of destruction within human cells (47), elicit immune responses.

Contrary to the low reactivity, phages tend to diminish inflammation caused by infection and other factors. Therefore, phages can repair tissues or improve other infection-related pathologies to eliminate the damage caused by excessive inflammation. For example, lysogenic Pf phages that infect *P. aeruginosa* frequently generate phage virions by induction within biofilms and can reduce lung injury by suppressing neutrophil recruitment, cytokine production, and phagocytosis (98). However, reducing inflammation does not automatically improve the disease state and may even diminish the antibacterial defense capabilities of hosts. Moreover, such effects are associated with decreased bacterial clearance, making chronic *P. aeruginosa* infections more difficult to treat and increasing mortality rates (98). Pf phages are endocytosed by white blood cells, and the RNA derived from their ssDNA is recognized by TLR3, which induces type I IFN production in a TRIF-dependent manner. Analyses using TRIF-deficient mice have shown that TNF production and phagocytosis are consequently suppressed. Furthermore, when Pf phage was added to bone marrow-derived DCs stimulated with LPS or alginate, TNF production was significantly suppressed (99). These results indicating immune suppressive effects of phage indicate that phages increase the risk of exacerbation of infections by excessive immunosuppression.

The intestinal tract is home to an immense number of phages—between 10^9^ and 10^10^ particles per gram of feces. They form symbiotic relationships with bacteria, reduce excessive inflammation, and modulate host immune system, thus aiding in maintaining ecological balance (100). As if to support that theory, serious adverse events related to phage therapy are exceedingly rare in clinical practice, and their safety and tolerability are considered high (101). Furthermore, its safety has been demonstrated in severe infectious diseases, such as infective endocarditis and septic shock (11). However, bacterial lysis by phage therapy results in the release of cell wall components, toxins, PAMPs, and DAMPs (102). As these components could theoretically trigger excessive inflammation and tissue damage, PRR-mediated inflammatory responses cannot be excluded (103). To address these complex issues, experimental systems and multivariate analyses are essential to distinguish the independent effects of factors such as phage type, route of administration, and host immune status (11, 101).

**Figure 2:**
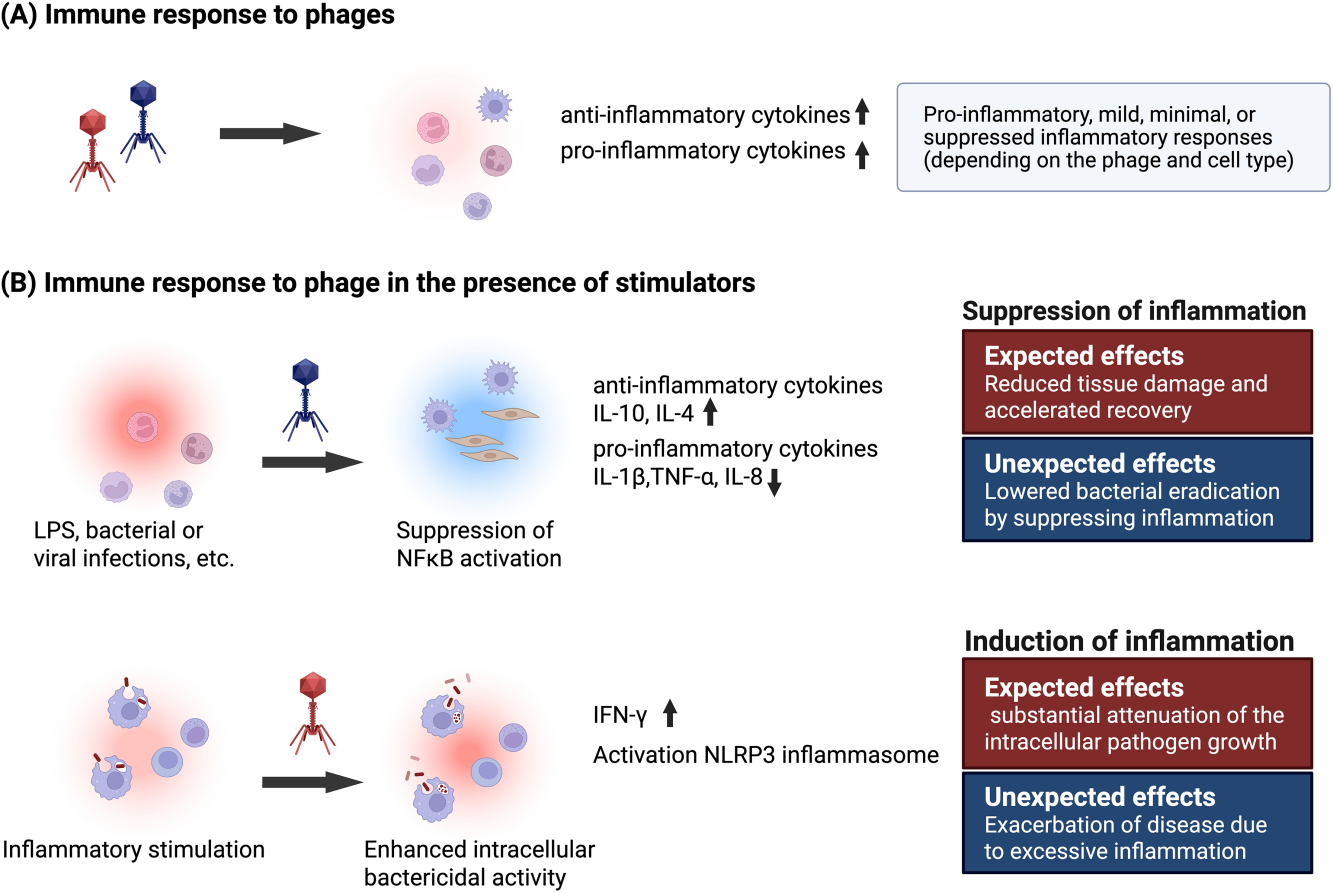
Anticipated dual nature of phages involved in eukaryotic immune system modulation on phage therapy. Severe inflammation has not been observed in response to the phage itself, although the response varies depending on the type of phage and the type of cell **(A)**. Conversely, in the presence of stimuli, either anti-inflammatory responses or the induction of inflammation may occur **(B)**. In cases of excessive inflammation induced by lipopolysaccharides (LPS), bacterial infection, or viral infection, certain phages upregulate the expression of anti-inflammatory cytokines and downregulate that of pro-inflammatory cytokines. This modulation helps to prevent severe tissue damage and ameliorate pathological conditions. However, there is a risk of exacerbating infections due to insufficient suppression of microbial proliferation. In contrast, if inflammation is induced by the phages, tissue damage may occur, worsening the condition. Nevertheless, the intracellular bactericidal mechanisms of phagocytes are enhanced, leading to the amelioration of infectious diseases. Most phages examined thus far have demonstrated anti-inflammatory effects. Created in BioRender.

In summary, immune modulation by phages is dual in nature, as it can contribute to both the enhancement of therapeutic effects and worsening of disease conditions (**Figure 2**). It is crucial to select the most suitable phage, based on the type of infectious bacteria and the nature of the infection. Moreover, the development of analytical frameworks that can accurately assess the quality and intensity of immune responses during treatment is essential to establish phage therapy as a reliable therapeutic approach.

Phages are microorganisms that have coexisted with eukaryotes and their bacterial flora throughout evolutionary history; therefore, eukaryotic hosts are constantly exposed to them (52, 104, 105). Through direct or indirect interactions, phages can induce anti- or pro-inflammatory immune responses (106). The findings of this review suggest that the host immune system may not simply recognize phages as pathogens to be eliminated, but rather as factors that modulate immune responses. Research on the interactions between phages and the host immune system is still in its early stages, similar to the studies on human viruses. In the future, a precise understanding of phage selection based on infectious bacteria and disease conditions, along with the corresponding immune responses, will enhance the stability and reproducibility of phage therapy, ultimately leading to the development of novel therapeutic strategies for treating infectious diseases.
